# Assessment of Radiologic Extranodal Extension Using Combinatorial Analysis of Nodal Margin Breakdown and Metastatic Burden in Oropharyngeal Cancer

**DOI:** 10.3390/cancers15133276

**Published:** 2023-06-21

**Authors:** Sungryeal Kim, Hannah Park, Se Hyun Yeou, Jin Roh, Yoo Seob Shin, Chul-Ho Kim, Eun Ju Ha, Jeon Yeob Jang

**Affiliations:** 1 Department of Otolaryngology, College of Medicine, Ajou University, Incheon 22332, Republic of Korea; bage777@gmail.com (S.K.); ostium@ajou.ac.kr (C.-H.K.); 2 Department of Otolaryngology, School of Medicine, Ajou University, Suwon 16502, Republic of Korea; doctorhannahpark@gmail.com (H.P.); scieth17@naver.com (S.H.Y.); ysshinmd@ajou.ac.kr (Y.S.S.); 3 Department of Pathology, School of Medicine, Ajou University, Suwon 16502, Republic of Korea; jin.roh327@gmail.com; 4 Department of Molecular Science and Technology, Graduate School of Medicine, Ajou University, Suwon 16502, Republic of Korea; 5 Department of Radiology, School of Medicine, Ajou University, Suwon 16502, Republic of Korea; 6 Department of Biomedical Sciences, Graduate School of Medicine, Ajou University, Suwon 16502, Republic of Korea

**Keywords:** oropharyngeal cancer, lymph node metastasis, extranodal extension

## Abstract

**Simple Summary:**

The presence of extranodal extension is a significant prognostic factor in oropharyngeal squamous cell carcinoma. Despite its significance as a prognostic factor, the predictive efficacy of preoperative ENE outcomes falls short of clinical expectations. The aim of this study was to examine the nodal margin-related feature and nodal tumor burden-related feature to enhance the predictive power of preoperative ENE. The study revealed that the inclusion of both margin and burden-related features resulted in higher predictive power compared with the use of either feature alone in predicting ENE. It would be beneficial to incorporate nodal tumor burden assessment in addition to nodal margin evaluation to enhance the accuracy of ENE prediction.

**Abstract:**

The importance of risk stratification in the management of oropharyngeal squamous cell carcinoma (OPSCC) is becoming increasingly obvious with the growing evidence of its variable prognosis. We identified and evaluated imaging characteristics predictive of extranodal extension (ENE) in OPSCC. Preoperative computed tomography and histopathologic results of 108 OPSCC patients who underwent neck dissection as primary treatment were analyzed. Imaging characteristics were reassessed for factors associated with nodal margin breakdown and metastatic burden. Moreover, the predictability of pathological ENE (pENE) was analyzed. Univariate and multivariate binomial logistic regression analyses were performed to examine the predictive power of ENE-related radiologic features. Imaging-based characteristics showed variable degrees of association with pENE. Factors associated with nodal margin breakdown (indistinct capsular contour, irregular margin, and perinodal fat stranding) and factors associated with nodal burden (nodal matting, lower neck metastasis, and presence of >4 lymph node metastases) were significantly predictive of ENE (odds ratio (OR) = 11.170 and 12.121, respectively). The combined utilization of the nodal margin and burden factors further increased the predictive ability (OR = 14.710). Factors associated with nodal margin breakdown and nodal burden were associated with pENE, demonstrating the use of combinatorial analysis for more accurate ENE prediction.

## 1. Introduction

Oropharyngeal squamous cell carcinoma (OPSCC), a subtype of head and neck cancer, may occur in the tonsils, base of the tongue, pharyngeal wall, and soft palate; it had a worldwide annual incidence of 98,412 cases in 2020 [1]. Major risk factors are human papilloma virus (HPV) infection and smoking, and the incidence rates of OPSCC and specifically HPV-associated OPSCC have increased since the 2000s [2]. HPV-associated OPSCC has better prognosis than non-HPV-associated disease, and OPSCC has been considered to have relatively good prognosis among head and neck cancers overall. However, the reported survival rates vary across studies, and significant proportions of patients still die because of this disease [3]. Thus, risk stratification is needed for the proper management of OPSCC.

Extranodal extension (ENE) is the growth of tumor cells beyond the capsule of a metastatic lymph node and into adjacent tissues. The presence of ENE is an important prognostic factor that is associated with an increased rate in disease recurrence and decreased survival [4,5]. The importance of ENE was reflected in the eighth tumor/node/metastasis (TNM) classification system by both the Union for International Cancer Control and the American Joint Committee on Cancer (AJCC) for non-viral-mediated OPSCC [6,7]. Several studies reported that the presence of ENE in HPV-mediated OPSCC affected the survival rate [8,9,10]. Therefore, the prediction of ENE before treatment is important for assessing prognosis and determining a therapeutic strategy.

There have been several studies aiming to predict ENE in head and neck cancer using preoperative imaging. Earlier studies attempted to predict ENE based on the shape of lymph node metastasis (LNM) from computed tomography (CT), magnetic resonance imaging, or ultrasonography [11,12,13]. Recently, there have been attempts to increase the predictive power by adding parameters from positron emission tomography/CT (PET/CT) or using machine learning [14,15]. In response to these efforts, several studies claimed that ENE detected by preoperative imaging, referred to as radiologic ENE (rENE), may be comparable with clinical TNM and other prognostic factors [16]. However, the pooled sensitivity and specificity of rENE remain limited and range from 75% to 79% [12,17], which suggests that further investigation is needed.

In this study, we hypothesized that besides the characterization of metastatic lymph node margins, additive analysis for the burden of LNM, as indicated by size and number, may further increase the predictive power of ENE. This study aimed to evaluate the relationship between the ENE status and characteristics of LNM on CT, including nodal margin and nodal burden-related features, to improve the predictive power of ENE.

## 2. Materials and Methods

### 2.1. Study Participants and Data Collection

The study protocol was approved by the Institutional Review Board of Ajou University Hospital (approval no. AJIRB-MED-MDB-21-418). This study included patients with OPSCC who were treated between January 2012 and May 2020 at Ajou University Hospital. The study included patients who (i) were diagnosed with OPSCC via biopsy, (ii) had preoperative CT scans, and (iii) underwent surgery including neck dissection as an initial treatment. The study excluded patients who underwent salvage surgery due to recurrence after initial treatment (surgery, radiotherapy, and concurrent chemoradiotherapy). The HPV status of each case of OPSCC was assessed by p16 immunohistochemical staining or the Cobas^®^ HPV test (Roche Molecular Diagnostics, Mannheim, DE, Germany) in accordance with the manufacturer’s instructions. A total of 108 patients with OPSCC were finally included (93 men and 15 women; mean age 59 years, range 52–67 years; Table 1).

### 2.2. Radiologic Image Acquisition and Analyses

All patients underwent preoperative CT scans for staging work-up. Preoperative CT images were obtained using 64- or 128-channel multi-detector scanners (SOMATOM Definition Flash, Siemens Medical Solutions, Cary, NC, USA; Brilliance, Philips Medical Systems, Best, The Netherlands). CT images were obtained at 0.5–0.6 mm collimation and were reconstructed into axial images every 3.0 mm on a 512 × 512 matrix. Pre-contrast and post-contrast CT scans with a 60 s scan delay after intravenous injection of 90 mL iodinated contrast agent were obtained. An experienced radiologist (E.J.H.; 15 years of experience in head and neck imaging) assessed and stratified the imaging features of cervical lymph nodes on CT scans in terms of the presence/absence of cervical LNM (>1 cm with loss of hilar fat or abnormal round shape), size of the largest metastasis (≤3 cm vs. >3 cm), number of metastases (≤2 vs. >2), location (ipsilateral vs. contralateral, retropharyngeal, or level IV/V), and nodal imaging features. The nodal imaging features were evaluated in terms of nodal margin, nodal matting, and presence of internal necrosis. Nodal margins were classified as indistinct capsular contours, irregular margins, perinodal fat stranding, or invasion into the adjacent structures as described in previous reports [18,19,20,21,22]. Nodal matting was regarded as a case in which three or more nodes were abutting such that the fat plane between them disappeared [23]. The radiologist was blinded to all other data including the final histological diagnoses.

### 2.3. Surgical Specimens and Evaluation of ENE

All patients underwent surgeries, including lymph node dissection. Fresh tissue samples were collected during surgery, and pathological diagnosis was made by experienced pathologists. The pathological information included tumor subsite, size, histologic grade, LNM, vascular invasion, perineural invasion, and TNM staging. When cervical LNMs were identified, the number of metastatic nodes involved, size (greatest dimension) of the largest metastatic focus in the lymph node, and ENE status were further analyzed. The TNM staging was defined according to the AJCC 8th edition.

### 2.4. Statistical Analysis

The chi-squared or Fisher’s exact test was used to compare categorical variables, and an independent *t*-test was used to compare continuous variables between the ENE-positive and ENE-negative groups. Sensitivity, specificity, positive predictive value (PPV), negative predictive value (NPV), and accuracy were used to analyze radiologic features. Univariate and multivariate binomial logistic regression analyses were performed to examine the predictive power of radiologic features with respect to ENE. All statistical analyses were performed using SPSS Version 18 (SPSS, Inc., Chicago, IL, USA), and a *p*-value < 0.05 was considered statistically significant.

## 3. Results

### 3.1. Clinicopathologic Characteristics of Patients with OPSCC with Respect to pENE

The clinicopathologic characteristics of patients with OPSCC were evaluated with respect to the pathological ENE (pENE) status (Table 1).

Among the 108 patients, 67 were pENE-positive (67/108, 62.0%) and 41 were pENE-negative (41/108, 38.0%). There were 76 HPV-positive patients, accounting for 70% of the total study population. There were no significant differences in age, sex, HPV status, smoking, pathologic T stage, tumor grade, lymphovascular invasion, or perineural invasion between the pENE-positive and pENE-negative groups. The pENE-positive group showed a significantly higher N classification and number of pathologically identified LNMs than the pENE-negative group (*p* < 0.001).

### 3.2. Radiologic Characteristics of LNM with Respect to pENE

We examined whether the characteristics of LNM, such as the node size, number, location, and morphology, were associated with pENE (Table 2). The presence of >4 LNMs and the occurrence of retropharyngeal or lower neck LNMs were significantly associated with the pENE-positive group (*p* < 0.001, *p* = 0.025, and *p* = 0.004, respectively). However, LNMs with a maximal diameter >3 cm (*p* = 0.524) or the presence of contralateral LNMs (*p* = 0.086) was not significantly associated with the pENE status. Next, we evaluated the association between CT features that are predictive of pENE in OPSCC. These features included indistinct capsular contour, irregular margin, perinodal fat stranding, invasion into the adjacent structures, nodal matting, and nodal necrosis and were carefully selected based on literature review [18,19,20,21,22]. The analyses in our cohort showed statistically significant associations of five of six parameters (indistinct capsular contour, irregular margin, perinodal fat stranding, invasion into the adjacent structures, and nodal matting) with pathological pENE (all *p* values < 0.05), while nodal necrosis was not significantly associated with pENE (*p* = 0.147).

### 3.3. Association of pENE Status with Radiological Features from Patients with OPSCC

A univariate and multivariate logistic regression analysis was performed to determine whether pENE could be predicted by radiologic features shown in Table 2 (Table 3).

Although the radiologic features, except retropharyngeal LNMs, significantly predicted pENE in univariate analysis, the seven radiologic features were not associated with pENE in multivariate analysis.

We divided the radiological features into two groups: nodal margin-related features and nodal burden-related features, assuming that the definitions of the CT imaging features were unclear, and the strong correlation between each feature resulted in insignificant results in the multivariate analysis. Indistinct capsular contour, irregular margins, perinodal fat stranding, and invasion into adjacent structures were considered as nodal margin-related features. The presence of >4 LNMs, lower neck LNM, and nodal matting were considered nodal burden-related features. Although the number of patients in the HPV-negative group was small, subgroup analysis was conducted for the relationship between pENE and related features according to the HPV status (Table 4).

**Figure 1:**
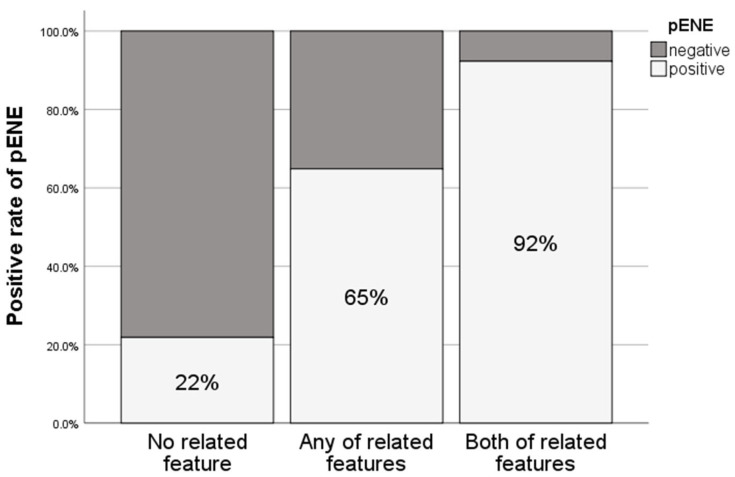
Association between the rates of pENE and presence of nodal margin- and burden-related features. pENE, pathologic extranodal extension.

In the HPV-positive group, all features were found to be related to pENE, but in the HPV-negative group, only irregular margins were found to be significant. The rate of pENE rose to 65% in the presence of either the margin- or burn-related features (Figure 1). The rate of pENE was further increased to 92% when margin- and burden-related features coexisted (Figure 1). The predictive power of related features was evaluated using univariate and multivariate logistic regression analyses in all patients (Table 5). The margin- or burden-related features as a single variable were significant in univariate analysis (with odds ratios of 11.170 and 12.121, respectively). Multivariate analysis also indicated the significant predictability of ENE by imaging-based characteristics. Importantly, the analysis of both related features further increased the predictability for pENE with an odds ratio of 14.710.

### 3.4. Diagnostic Performance of Radiological Features for Predicting pENE of Cervical LNMs from Patients with OPSCC

Sensitivity, specificity, PPV, NPV, and accuracy of radiologic features were calculated in all patients and the HPV-positive group (Table 6).

Overall, nodal margin-related features showed high sensitivity but low specificity, while nodal burden-related features had low sensitivity but relatively high specificity (86.6% vs. 56.7% and 63.4% vs. 90.2%, respectively). The sensitivity of both related features was lower than that of each related feature, but the specificity was higher than that of each related feature (53.7% and 92.7%, respectively). Similar results pertaining to each group of related features were observed in the HPV-positive group (89.1% vs. 56.5% and 63.3% vs. 86.7%, respectively). The sensitivity and specificity of both related features in the HPV-positive group showed the same pattern (54.3% and 90.0%, respectively).

## 4. Discussion

This study assessed the association between CT characteristics and pENE status. Although both nodal margin- and burden-related features on CT scans showed significant associations with the ENE status, the former tended to have high sensitivity (86.6%) and low specificity (63.4%), while the latter showed a relatively low sensitivity (56.7%) and high specificity (90.2%). The combinatorial analysis of both margin- and burden-related features increased the ability to predict pENE.

The rENE has been shown to be associated with poor prognosis in head and neck cancer [5,24,25,26]. A Canadian group reported an increased rate in distant metastasis with decreased overall survival when rENE was present in OPSCC [5,25]. Kim et al. performed a different treatment protocol such that primary concurrent chemoradiotherapy was conducted in the presence of rENE in early-stage HPV-positive OPSCC, while primary surgery was performed in the absence of rENE. There was no difference in the survival or locoregional recurrence rates between the two groups [27]. In addition, the National Comprehensive Cancer Network guidelines mentioned that concurrent chemoradiation therapy is preferred for those with clinical evidence of fixed or matted nodes or obvious extranodal extension in patients with p16-positive OPSCC staged T0-2/N1, T0-2/N2, or T3/N0-2 [28].

Several studies have used different variables to predict pENE. In these studies, since the definitions of the morphological characteristics of the metastatic lymph node and its relationship with surrounding tissues to predict pENE were not clear, the presence or absence of the characteristics varied depending on the radiologist. In a study by Fataji et al., inter-rater agreement was high for nodal necrosis and matting, which were clearly defined, but low for irregular margins and indistinct capsular contours [18]. Moreover, despite the relatively objective measuring of the metastatic lymph nodes sizes, these were inconsistently estimated by different radiologists in the same study [21,29]. There may be limitations in predicting pENE using a single characteristic because morphological characteristics and the relationship with the surrounding tissues can be differently evaluated depending on the radiologist’s proficiency. A consensus on a clear definition is required to predict ENE with these variables.

The number and location of LNMs are considered as variables to evaluate the burden of metastatic lymph nodes [30] and have been used as variables for pENE prediction in several studies. Regarding the number of lymph nodes, An et al. reported that pENE was associated with a higher number of pathologically involved lymph nodes (2.2 vs. 3.9 involved nodes) [31]. Geltzeiler et al. showed that the rate of pENE was significantly higher with three or more radiographically suspicious nodes [32]. The locations of LNMs have also been evaluated to predict pENE. Joo et al. and Huang et al. reported that retropharyngeal LNMs were associated with pENE [19,25]. Geltzeiler et al. evaluated level 4 and contralateral LNMs as variables for predicting pENE [32]. According to the TNM staging of HPV-positive OPSCC, we divided patients into two groups: those with >4 LNMs and those with ≤4 LNMs. Our result showed that the number of LNMs was associated with ENE. Unlike other studies, retropharyngeal LNMs cannot predict pENE, but lower neck LNMs can. However, poor prognosis has been reported when LNMs appeared in the contralateral side of the retropharynx or lower neck; thus, it is thought that the location of LNMs should be considered in future research [33,34,35].

Considering that (i) there are no clear criteria for using nodal morphology or nodal burden to predict pENE and (ii) nodal burden also contributes to pENE in a different way than nodal morphology, we classified several indices into margin- or burden-related features. Our results revealed that each of these related features were related to pENE, and the predictive power was improved when two related features coexisted.

This study had several limitations. First, this was a retrospective study conducted at a single institution. Additional validation study in a new clinical cohort is warranted in the future. Second, this study assessed the presence of pENE, but not its degree, as an analytic parameter. We performed further analysis using 27 pathologically available specimens to differentiate major (>2 mm) and minor (≤2 mm) ENE. Preliminary analysis showed variable degree of associations between margin- or burden-related features and the presence of major or minor ENE (Supplementary Table S1). However, these results were obtained using a small sample size; thus, we will collect more data in the future to conduct further research. Third, this study did not incorporate another imaging modality. Maximum standardized uptake value, total lesion glycolysis, and metabolic tumor volume on PET/CT have been recently used to estimate metastatic lymph node burden and ENE. However, the cut-off values for ENE prediction using these parameters vary among studies [14,36,37,38], and these parameters are considered more meaningful than others because of their objective and numerical value despite the lack of consensus.

## 5. Conclusions

In conclusion, our study analyzed preoperative CT findings of patients with OPSCC to evaluate the relationship between pENE and characteristics of LNM. The results revealed that not only the shape of LNMs, a commonly used feature, but also the presence of >4 LNMs and localization in the lower neck were associated with pENE. Moreover, classifying the characteristics of LNM into margin- and burden-related features increased the predictive power for pENE. Our study suggests that a holistic approach for predicting ENE, including the classification of the characteristics of LNM, reduces the discrepancy between rENE and pENE, and further studies on rENE will help in risk stratification for patients with OPSCC.

## Figures and Tables

**Table 1:** Clinicopathological characteristics of the patients in relation to ENE status (N = 108).

	Total N = 108 No. of Patients (%)	pENE (−), N = 41 No. of Patients (%)	pENE (+), N = 67 No. of Patients (%)	*p*-Value *^a^*
Clinical characteristics				
Age (years)				0.591
Mean, [25–75th percentile]	59 (52–67)	59 (49–67)	60 (53–67)	
Sex				0.454
Men	93 (86)	34 (83)	59 (88)	
Women	15 (14)	7 (17)	8 (12)	
HPV status				0.894
Negative	24 (22)	8 (20)	16 (24)	
Positive	76 (70)	30 (73)	46 (69)	
Unknown	8 (8)	3 (7)	5 (7)	
Smoking history				0.796
Non-smoker	46 (43)	15 (37)	31 (46)	
Current Smoker	32 (30)	13 (32)	19 (28)	
Ex-smoker	27 (25)	12 (29)	15 (23)	
Unknown	3 (2)	1 (2)	2 (3)	
N classification				0.001
N0	6 (6)	6 (15)	0 (0)	
N1	70 (64)	29 (70)	41 (61)	
N2–N3	32 (30)	6 (15)	26 (39)	
Pathologic characteristics				
Tumor subsite				0.546
Palatine tonsil	91 (85)	35 (88)	56 (84)	
Base of tongue	12 (11)	3 (7)	9 (13)	
Others	4 (4)	2 (5)	2 (3)	
T classification				0.438
Metastasis of unknown primary	1	1 (3)	0	
T1–T2	71 (66)	27 (68)	43 (64)	
T3–T4	36 (34)	12 (29)	24 (36)	
Tumor grade				0.604
Well differentiated	5 (5)	1 (3)	4 (6)	
Moderately differentiated	57 (53)	19 (47)	38 (57)	
Poorly differentiated	32 (30)	14 (35)	19 (27)	
Unknown	13 (12)	6 (15)	7 (10)	
Lymphovascular invasion				0.191
Absent	45 (42)	21 (52)	24 (36)	
Present	43 (40)	12 (30)	31 (46)	
Unknown	19 (18)	7 (18)	12 (18)	
Perineural invasion				0.715
Absent	76 (71)	30 (75)	46 (69)	
Present	11 (10)	3 (8)	8 (12)	
Unknown	20 (19)	7 (17)	13 (19)	
Pathologically identified LNs				<0.001
0	4 (4)	4 (10)	0 (0)	
≤4	75 (69)	34 (83)	41 (61)	
>4	29 (27)	3 (7)	26 (39)	

pENE: pathologic extranodal extension; *^a^*: statistical analyses between ENE-positive vs. ENE-negative.

**Table 2:** Radiological characteristics of LNM in relation to ENE.

	Total = 108 No. of Patients (%)	pENE (−) No. of Patients (%)	pENE (+) No. of Patients (%)	*p*-Value *^a^*
Preoperative imaging characteristics of lymph node metastasis (LNM)				
Maximum diameter (cm)				0.333
≤3 cm	73 (68)	30 (73)	43 (64)	
>3 cm	35 (32)	11 (27)	24 (36)	
Number of LNM				<0.001
0	6 (6)	6 (15)	0 (0)	
≤4	76 (70)	33 (81)	44 (64)	
>4	26 (24)	2 (4)	24 (36)	
Retropharyngeal LNM				0.025
No	99 (92)	40 (98)	59 (88)	
Yes	9 (8)	1 (2)	8 (12)	
Lower neck (level IV or V) LNM				0.004
No	88 (81)	39 (95)	49 (73)	
Yes	20 (19)	2 (5)	18 (27)	
Contralateral LNM				0.086
No	98 (91)	39 (97)	59 (87)	
Yes	10 (9)	1 (3)	9 (13)	
Indistinct capsular contour				<0.001
No	36 (33)	27 (66)	9 (13)	
Yes	72 (67)	14 (34)	58 (87)	
Irregular margin				<0.001
No	62 (57)	35 (85)	27 (40)	
Yes	46 (43)	6 (15)	40 (60)	
Absence of perinodal fat plane				<0.001
No	48 (44)	33 (81)	15 (22)	
Yes	60 (56)	8 (19)	52 (78)	
Invasion into ST				0.001
No	89 (82)	40 (98)	49 (73)	
Yes	19 (18)	1 (2)	18 (27)	
Nodal matting				<0.001
No	77 (71)	40 (98)	37 (55)	
Yes	31 (29)	1 (2)	30 (45)	
Nodal necrosis				0.147
No	26 (21)	13 (32)	13 (19)	
Yes	82 (79)	28 (68)	54 (81)	

pENE: pathologic extranodal extension; *^a^*: statistical analyses between ENE-positive vs. ENE-negative groups; LNM: lymph node metastasis; ST: surrounding tissue.

**Table 3:** Logistic regression of CT imaging findings.

	Total = 108 No. of Patients (%)	Univariate Analysis OR [95% CI]	*p*-Value	Multivariate Analysis OR [95% CI]	*p*-Value
Characteristics of LNM					
No. of LNM > 4	26 (24%)	10.884 [2.413–49.081]	0.002	1.340 [0.185–9.712]	0.772
Retropharyngeal LNM	9 (8%)	5.424 [0.653–45.062]	0.118	-	-
Lower neck LNM	20 (19%)	7.163 [1.566–32.759]	0.011	3.042 [0.375–24.677]	0.298
CT imaging features					
Indistinct capsular contour	72 (66%)	12.429 [4.788–32.259]	<0.001	2.493 [0.577–10.770]	0.221
Irregular margin	46 (43%)	8.642 [3.198–23.354]	<0.001	0.910 [0.200–4.146]	0.903
Absence of perinodal fat plane	60 (56%)	14.300 [5.461–37.444]	<0.001	3.646 [0.728–18.260]	0.115
Invasion into ST	19 (18%)	14.694 [1.879–114.888]	0.010	1.715 [0.151–19.489]	0.664
Nodal matting	31 (28%)	32.432 [4.209–249.900]	0.001	8.023 [0.861–74.797]	0.068

CT: computed tomography; OR: odds ratio; CI: confidence interval; LNM: lymph node metastasis; ST: surrounding structures.

**Table 4:** Subgroup analysis of CT imaging findings of lymph node metastasis for detecting pENE in OPSCC.

	HPV + Patients			HPV − Patients		
	Total = 76 No. of Patients (%)	pENE (+) = 46 No. of Patients (%)	*p*-Value	Total = 24 No. of Patients (%)	pENE (+) = 16 No. of Patients (%)	*p*-Value
Margin-related feature (any of the 4)	52 (68)	41 (89)	<0.001	15 (63)	12 (75)	0.099
Indistinct capsular contour	51 (67)	41 (89)	<0.001	15 (63)	12 (75)	0.099
Irregular margin	30 (40)	26 (57)	<0.001	11 (46)	10 (63)	0.033
Absence of perinodal fat plane	41 (54)	36 (78)	<0.001	13 (54)	11 (69)	0.082
Invasion into ST	13 (17)	12 (26)	0.010	6 (25)	6 (38)	0.066
Burden-related feature (any of the 3)	30 (40)	26 (57)	<0.001	9 (38)	9 (56)	0.009
Lower neck LNM	14 (18)	12 (26)	0.033	5 (21)	5 (31)	0.130
Nodal matting	24 (32)	23 (50)	<0.001	5 (21)	5 (31)	0.130
No. of LNM >4	17 (22)	15 (33)	0.008	6 (25)	6 (38)	0.066

LNM: lymph node metastasis; ST: surrounding tissue; pENE: pathologic extranodal extension; OPSCC: oropharyngeal squamous cell carcinoma; HPV: human papilloma virus; CT: computed tomography.

**Table 5:** Logistic regression of groups of CT imaging findings at all patients.

All Patients	Univariate Analysis OR [95% CI]	*p*-Value	Multivariate Analysis OR [95% CI]	*p*-Value
Margin-related feature	11.170 [4.333–28.798]	<0.001	6.466 [2.354–17.759]	<0.001
Burden-related feature	12.121 [3.880–37.868]	<0.001	6.677 [1.993–22.369]	0.002
Both related features	14.710 [4.132–52.365]	<0.001		

OR: odds ratio; CI: confidence interval; CT: computed tomography.

**Table 6:** Diagnostic performance of CT imaging findings of lymph node metastasis for detecting ENE in OPSCC.

	Sensitivity No. of Patients (%)	Specificity No. of Patients (%)	PPV No. of Patients (%)	NPV No. of Patients (%)	Accuracy No. of Patients (%)
**All**					
Margin-related feature (any of under 4)	58 (86.6)	26 (63.4)	58 (79.5)	26 (74.3)	84 (77.8)
Indistinct capsular contour	58 (86.6)	27 (65.9)	58 (80.6)	27 (75.0)	85 (78.7)
Irregular margin	40 (59.7)	35 (85.4)	40 (87.0)	35 (56.5)	75 (69.4)
Absence of perinodal fat plane	52 (77.6)	33 (80.5)	52 (86.7)	33 (68.8)	85 (78.7)
Invasion into ST	18 (26.9)	40 (97.6)	18 (94.7)	40 (44.9)	58 (53.7)
Burden-related feature (any of under 3)	38 (56.7)	37 (90.2)	38 (90.5)	37 (56.1)	75 (69.4)
Lower neck LNM	18 (26.9)	39 (95.1)	18 (90.0)	39 (44.3)	57 (52.8)
Nodal matting	30 (44.8)	40 (97.6)	30 (96.8)	40 (51.9)	70 (64.8)
No. of LNM > 4	24 (35.8)	39 (95.1)	24 (92.3)	39 (47.6)	63 (58.3)
Any of margin- or burden-related features	59 (88.1)	25 (61.0)	59 (78.7)	25 (75.8)	84 (77.8)
Both related features	36 (53.7)	38 (92.7)	36 (92.3)	38 (55.1)	74 (68.5)
**HPV (+) OPSCC**					
Margin-related feature (any of under 4)	41 (89.1)	19 (63.3)	41 (78.8)	19 (79.2)	60 (78.9)
Indistinct capsular contour	41 (89.1)	20 (66.7)	41 (80.4)	20 (80.0)	61 (80.3)
Irregular margin	26 (56.5)	26 (86.7)	26 (86.7)	26 (56.5)	52 (68.4)
Absence of perinodal fat plane	36 (78.3)	25 (83.3)	36 (87.8)	25 (71.4)	61 (80.3)
Invasion into ST	12 (26.1)	29 (96.7)	12 (92.3)	29 (46.0)	41 (53.9)
Burden-related feature (any of under 3)	26 (56.5)	26 (86.7)	26 (86.7)	26 (56.5)	52 (68.4)
LNM at Lv4	12 (26.1)	28 (93.3)	12 (85.7)	28 (45.2)	40 (52.6)
Nodal matting	23 (50.0)	29 (96.7)	23 (95.8)	29 (55.8)	52 (68.4)
No. of LNM > 4	15 (32.6)	28 (93.3)	15 (88.2)	28 (47.5)	43 (56.6)
Any of margin- or burden-related features	41 (89.1)	18 (60.0)	41 (77.4)	18 (78.3)	59 (77.6)
Both related features	25 (54.3)	27 (90.0)	25 (89.3)	27 (56.3)	52 (68.4)

OPSCC: oropharyngeal squamous cell carcinoma; PPV: positive predictive value; NPV: negative predictive value; LNM: lymph node metastasis; ST: surrounding tissue; CT: computed tomography; ENE: extranodal extension.

## Data Availability

The datasets generated and/or analyzed during the current study are not publicly available due to containing personally identifiable information but are available from the corresponding author on reasonable request.

## Supplementary Materials

The following supporting information can be downloaded at: https://www.mdpi.com/article/10.3390/cancers15133276/s1. Table S1. Logistic regression for degree of ENE at all patients.
