# Supplementary material for: Assessment of Radiologic Extranodal Extension Using Combinatorial Analysis of Nodal Margin Breakdown and Metastatic Burden in Oropharyngeal Cancer
Source: Cancers (Basel). 2023 Jun 21;15(13):3276. doi: 10.3390/cancers15133276 (PMC10339939; doi:10.3390/cancers15133276)
Supplement: Supplementary file 1 [file cancers-15-03276-s001.zip › cancers-2448524-supplementary.pdf]

Supplementary Table S1. Logistic regression for degree of ENE at all patients

**Minor ENE**	**Univariate analysis OR [95% CI]**	***P-value***	**Multivariate analysis OR [95% CI]**	***P-value***
Margin-related feature	3.706 [0.842-16.319]	0.083	1.290 [0.191-8.704]	0.794
Burden-related feature	9.500 [2.056-43.888]	**0.004**	8.187 [1.261-53.168]	**0.028**
Both related features	11.700 [2.432-56.283]	**0.002**		
**Major ENE**	**Univariate analysis OR [95% CI]**	***P-value***	**Multivariate analysis OR [95% CI]**	***P-value***
Margin-related feature	11.912 [2.416-58.724]	**0.002**	8.808 [1.703-45.561]	**0.009**
Burden-related feature	5.630 [1.559-20.326]	**0.008**	3.054 [0.756-12.336]	0.117
Both related features	5.460 [1.427-20.884]	**0.013**		

OR: Odds ratio, CI: confidence interval, ENE: extranodal extension.
